# Identification of distinct SET/TAF-Iβ domains required for core histone binding and quantitative characterisation of the interaction

**DOI:** 10.1186/1471-2091-10-10

**Published:** 2009-04-09

**Authors:** Zoe Karetsou, Anastasia Emmanouilidou, Ioannis Sanidas, Stamatis Liokatis, Eleni Nikolakaki, Anastasia S Politou, Thomais Papamarcaki

**Affiliations:** 1 Laboratory of Biological Chemistry, Medical School, University of Ioannina, 451 10 Ioannina, Greece; 2 Laboratory of Biochemistry, Department of Chemistry, Aristotle University of Thessaloniki, 541 24 Thessaloniki, Greece; 3 Foundation for Research and Technology-Hellas/Biomedical Research Institute, 451 10 Ioannina, Greece

## Abstract

**Background:**

The assembly of nucleosomes to higher-order chromatin structures is finely tuned by the relative affinities of histones for chaperones and nucleosomal binding sites. The myeloid leukaemia protein SET/TAF-Iβ belongs to the NAP1 family of histone chaperones and participates in several chromatin-based mechanisms, such as chromatin assembly, nucleosome reorganisation and transcriptional activation. To better understand the histone chaperone function of SET/TAF-Iβ, we designed several SET/TAF-Iβ truncations, examined their structural integrity by circular Dichroism and assessed qualitatively and quantitatively the histone binding properties of wild-type protein and mutant forms using GST-pull down experiments and fluorescence spectroscopy-based binding assays.

**Results:**

Wild type SET/TAF-Iβ binds to histones H2B and H3 with K_d _values of 2.87 and 0.15 μM, respectively. The preferential binding of SET/TAF-Iβ to histone H3 is mediated by its central region and the globular part of H3. On the contrary, the acidic C-terminal tail and the amino-terminal dimerisation domain of SET/TAF-Iβ, as well as the H3 amino-terminal tail, are dispensable for this interaction.

**Conclusion:**

This type of analysis allowed us to assess the relative affinities of SET/TAF-Iβ for different histones and identify the domains of the protein required for effective histone recognition. Our findings are consistent with recent structural studies of SET/TAF-Iβ and can be valuable to understand the role of SET/TAF-Iβ in chromatin function.

## Background

The genomes of eukaryotic cells are organized as chromatin, a highly dynamic complex between DNA and histones. The fundamental repeating unit in chromatin is the nucleosome core particle (NCP), which is composed of 147 bp of DNA wrapped around an octameric core containing two copies of each of the four histones H2A, H2B, H3, and H4 [1]. Ultimately, long arrays of nucleosomes are further compacted into higher-order structures that form functional chromatin domains. The packaging of DNA into chromatin ensures genome stability, but at the same time creates a barrier for the access of cellular factors to underlying DNA sequences, thus influencing gene expression, DNA replication, recombination and repair [2]. The major mechanisms which modulate chromatin structure include ATP-dependent nucleosome remodeling complexes, covalent modification of histones, histone variant exchange and histone chaperones [3]. ATP-dependent remodeling complexes use the energy of ATP hydrolysis to regulate chromatin fluidity, leading to either activation or repression of transcription [4,5]. Recent studies have revealed a large number of post-translational modifications that lie either in the flexible histone tails or in the histone globular domain, which are correlated with distinct functional states of chromatin [6-8]. In concert to these machineries, non-histone proteins and histone chaperones regulate nucleosome dynamics by influencing the binding affinity of histones for DNA [9].

Histone chaperones are key players in the organisation of chromatin domains [9-11]. They shield the positive charge of histones and safeguard them from improper electrostatic interactions with DNA or other proteins which could be harmful for the cell. Consequently, they mediate chromatin assembly/disassembly, histone storage and coordinate the exchange/deposition of histones onto DNA during replication. The best characterised chaperones are NAP1 [12,13], chromatin assembly factor 1 (CAF1) [14], N1/N2 [15], nucleoplasmin [16], anti-silencing factor (ASF1) [17] and HIRA [18]. Despite the fact that all chaperones bind to histones, they seem to mediate distinct protein-protein interactions *in vivo *and their histone partners and binding affinities vary considerably.

The myeloid leukaemia protein SET/TAF-1β studied here, also known as I_2_PP2A, and INHAT, belongs to the NAP1 family of histone chaperones. It was originally identified as a translocated gene in acute undifferentiated leukemia [19-21] and is widely expressed in human and mouse tissues [22]. The analysis of SET/TAF-Iβ functions is challenging, because it is involved in a broad range of cellular mechanisms and it remains unclear how it switches between these different functions. SET/TAF-Iβ is a potent inhibitor of phosphatase 2A (PP2A), and interacts with several proteins involved in the regulation of cell cycle, such as p21waf1 [23], cyclin E-CDK2 [23], B- cyclins [24] and p35nck5a [25]. Furthermore, SET/TAF-Iβ has been identified as a key factor of the cytotoxic T lymphocytes (CTL)-induced cell death, suggesting its role in the regulation of apoptosis [26]. Besides its involvement in the control of cell cycle and apoptosis, increasing number of reports describe the chromatin-related properties of SET/TAF-Iβ. It stimulates DNA replication and transcription of the adenovirus genome [27-30] and induces chromatin decondensation [31]. Other studies have shown that SET/TAF-Iβ binds to nucleosomal histones [32-34] and inhibits histone acetylation by masking histone tails as a component of the INHAT complex [35]. In addition, we have previously reported the interaction of SET/TAF-Iβ with the chromatin remodeling protein prothymosin α [36] and the transcription coactivator CREB-binding protein (CBP) [37]. Consistent with the above studies, a DNA-microarray based analysis revealed that SET/TAF-Iβ stimulates the transcription of a sub-set of genes [38]. Interestingly, the transcription of the endogenous genes which were upregulated by SET/TAF-Iβ was found to be additively stimulated by histone acetylation. Recently, Ichijo et al [39] showed that Set/TAF-Iβ interacts with the activated glucocorticoid receptor and acts as a ligand-activated GR-responsive transcriptional repressor, which further suggests the involvement of this protein in transcriptional regulation.

Inspection of the aminoacid sequence of SET/TAF-Iβ reveals three interesting structural features, which are important determinants of its ligand binding activity and, ultimately, of its functional specificity. First, a motif located in the N-terminal region (amino acids 1–76) predicted to form a coiled-coil structure, which is believed to mediate the dimerisation of the protein [40]. Dimerisation is a conserved feature of the NAP1 family of histone chaperones and it was found to be necessary for the chromatin remodeling activity of SET/TAF-Iβ [40]. Second, the central NAP domain (amino acids 80–225), which is highly conserved among the NAP1 protein family members and is thought to mediate histone fold-specific binding [12,13]. Third, a highly acidic polyglutamic track located in the C-terminal tail of SET/TAF-Iβ (amino acids 226–277). This region consists of 42 glutamic and aspartic residues occasionally interspersed by glycine residues and seems to adopt an intrinsically disordered structure that provides structural plasticity, according to recent CD and X-Ray data on yeast NAP1 [12,41]. In vitro experiments suggested that the acidic tail might be implicated in the chaperone activity of SET/TAF-Iβ, targeting the basic N-terminal tails of histones [31,42]. However, it is still unclear which domains of the protein are required for effective histone recognition. In addition, the relative affinity of SET/TAF-Iβ for the various histones remains unknown. This information can be very valuable to understand the role of SET/TAF-Iβ as a histone chaperone, since histone binding activity is the common feature of this class of proteins, through which they are involved in a wide range of mechanisms, besides nucleosome assembly/disassembly, such as histone variant exchange, histone modification regulation, histone shuttling and storage and transient histone overload buffering [43,44]. Moreover, it has been recently shown that the assembly of nucleosomes to the level of chromatin is governed by affinity differences of histones between chaperones and binding sites on subnucleosomal complexes [41,45].

Here, we focused on the histone-binding properties of SET/TAF-Iβ. We performed GST-pull down analysis with wild type SET/TAF-Iβ and all five histones and fluorescence spectroscopy-based quantitative binding assays with those histones that appeared to interact with SET/TAF-Iβ. This analysis allowed us to assess the relative affinity of SET/TAF-Iβ for different histones. Such detailed analysis has not, to our knowledge, been carried out for this protein. Furthermore, in order to identify the regions of the protein responsible for the interaction with histones, we designed several SET/TAF-Iβ truncations, examined their structural integrity by circular dichroism and assessed qualitatively and quantitatively their histone-binding properties by GST-pull down assays and fluorescence spectroscopy-based titration experiments. Collectively, our data show that SET/TAF-Iβ binds preferentially to histone H3 through its central region. The dimerisation domain of SET/TAF-Iβ, as well as its C-terminal acidic tail, are not essential for this interaction.

## Results

### Preferential binding of of SET/TAF-Iβ to histone H3

**Figure 1:**
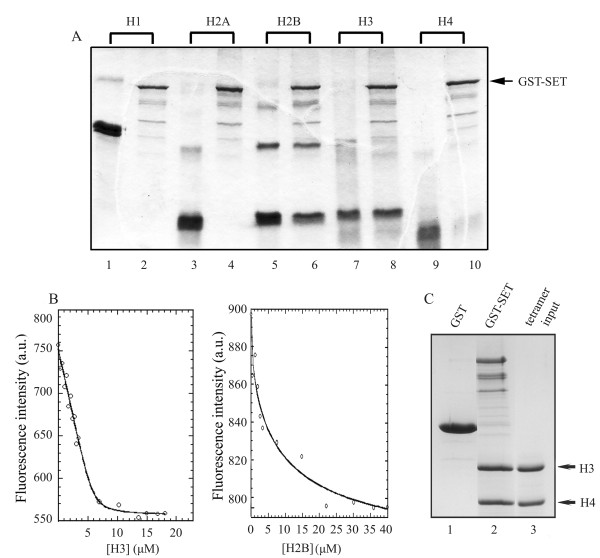
**Histone binding properties of SET/TAF-Iβ**. (A) *SET/TAF-Iβ binding to histones*. Purified GST-SET/TAF-Iβ (2 μg) was immobilised on glutathione-agarose beads and incubated with 4 μg of H1 (lane 2) or core histones H2A, H2B, H3, H4 (lanes 4, 6, 8, 10, respectively) as indicated in Materials and Methods. The beads were washed and analysed by SDS-PAGE and Coomassie blue staining. Lanes 1, 3, 5, 7, 9 indicate the input of the histones. (B) *Fluorescence-based binding assays*. SET/TAF-Iβ at an initial concentration of 4 μM was titrated with increasing amounts of purified calf thymus H3 (left panel) or H2B (right panel) stock solutions in PBS buffer. SET/TAF-Iβ binding to histones H3 and H2B was followed by monitoring the intensity of the fluorescence emitted at 345 nm after excitation at 295 nm. The solid line represents the binding curve derived by non-linear regression analysis of the fluorescence values corrected for dilution and for buffer contribution. (C) *SET/TAF-Iβ binding to (H3/H4)2 tetramers*. GST (lane 1) and GST-SET/TAF-Iβ (lane 2) were immobilised on glutathione-agarose beads and incubated with reconstituted (H3/H4)_2 _tetramers (lane 3) as indicated in Materials and Methods. The beads were washed and bound proteins were analysed by SDS-PAGE and Coomassie blue staining.

To study the histone binding activity of *SET/TAF-Iβ*, we performed GST-pull down assays. GST-SET/TAF-Iβ was immobilised on glutathione beads and challenged with H1 or core histones at high salt concentrations. The results revealed binding of SET/TAF-Iβ to histones H2B (Fig. 1A, lane 6) and H3 (lane 8), while no interaction was observed with histones H1 (lane 2), H2A (lane 4) and H4 (lane 10). GST immobilized on glutathione beads was used as a negative control (not shown).

To compare quantitatively the relative affinity of SET/TAF-Iβ for histones H3 and H2B, we performed fluorescence-based titration experiments [46,47]. SET/TAF-Iβ sequence contains four tryptophan residues, which could serve as intrinsic fluorescence chromophores. On the other hand, histones are poor in aromatic amino acids and completely devoid of tryptophan residues. Therefore, the binding affinity of SET/TAF-Iβ to histones H3 and H2B could be monitored by fluorescence spectroscopy at an excitation wavelength that precludes a contribution to the emitted fluorescence intensity from the tyrosine residues of histones. A clear and gradual quenching of the fluorescence signal was observed, when increasing amounts of histones were added to full-length SET/TAF-Iβ. The fluorescence intensity change was used for a quantitative binding assay. A binding curve was obtained by monitoring the intensity at 345 nm of the fluorescence emitted by SET/TAF-Iβ as a function of added histone concentration at constant temperature (Fig. 1B). Fitting of the fluorescence data resulted in a K_d _value of 0.15 μM for the SET/TAF-Iβ-H3 interaction and a K_d _value of 2.87 μM for the SET/TAF-Iβ-H2B interaction (Table 1). The qualitative and quantitative assessment of the SET/TAF-Iβ-histone interaction points to a clear preference of SET/TAF-1β for histone H3. Pull-down experiments with GST-fused SET/TAF-Iβ and reconstituted (H3/H4)_2 _tetramers showed that this interaction is also efficient in the more "organized" context of the (H3/H4)_2 _tetramer (Fig. 1C). In this sense, SET/TAF-Iβ behaves similarly to NAP1 from yeast, Drosophila and humans which have been shown to preferentially bind the (H3/H4)_2 _tetramer *in vitro *[41], although NAP family members have generally been described as H2A/H2B chaperones. On the contrary, yeast NAP1 is capable of binding to linker histone H1 [48], while our data rule out such a possibility for SET/TAF-Iβ (Fig. 1A, lane 2). NAP1 from yeast and humans has also been shown to facilitate the removal of H2A-H2B dimers from a folded nucleosome [49,50], but in the same set of experiments, SET/TAF-Iβ did not demonstrate an efficient stripping activity of the dimer [49]. This difference together with the clear preference of SET/TAF-Iβ for H3 binding and with the lack of interaction with linker histone H1 may account, at least in part, for distinct roles of SET/TAF-Iβ in diverse mechanisms of nucleosome assembly and reorganization.

**Table 1:** Relative affinities of SET-TAFI-β and its deletion mutants for H3

**SET/TAF-Iβ construct**	**K_d_**	**Pull-down results**
SET 1–277	0.15 ± 0.20	+
SET 1–210	0.13 ± 0.10	+
SET 76–210	0.14 ± 0.08	+
SET 76–185	0.10 ± 0.08	+
SET 110–277	0.16 ± 0.12	+
SET 110–210	n.d.	-

Binding of the various SET/TAF-Iβ deletion mutants with histone H3 was followed by monitoring the intensity of the fluorescence emitted by the tryptophan residues of SET/TAF-Iβ polypeptides upon addition of increasing amounts of calf thymus histone H3. The dissociation constant (*K*_*d*_) for the complexes formed was estimated by non-linear regression analysis (KaleidaGraph, Synergy Software), as indicated in Methods.

### Individual domains of SET/TAF-Iβ are sufficient for histonebinding

**Figure 2:**
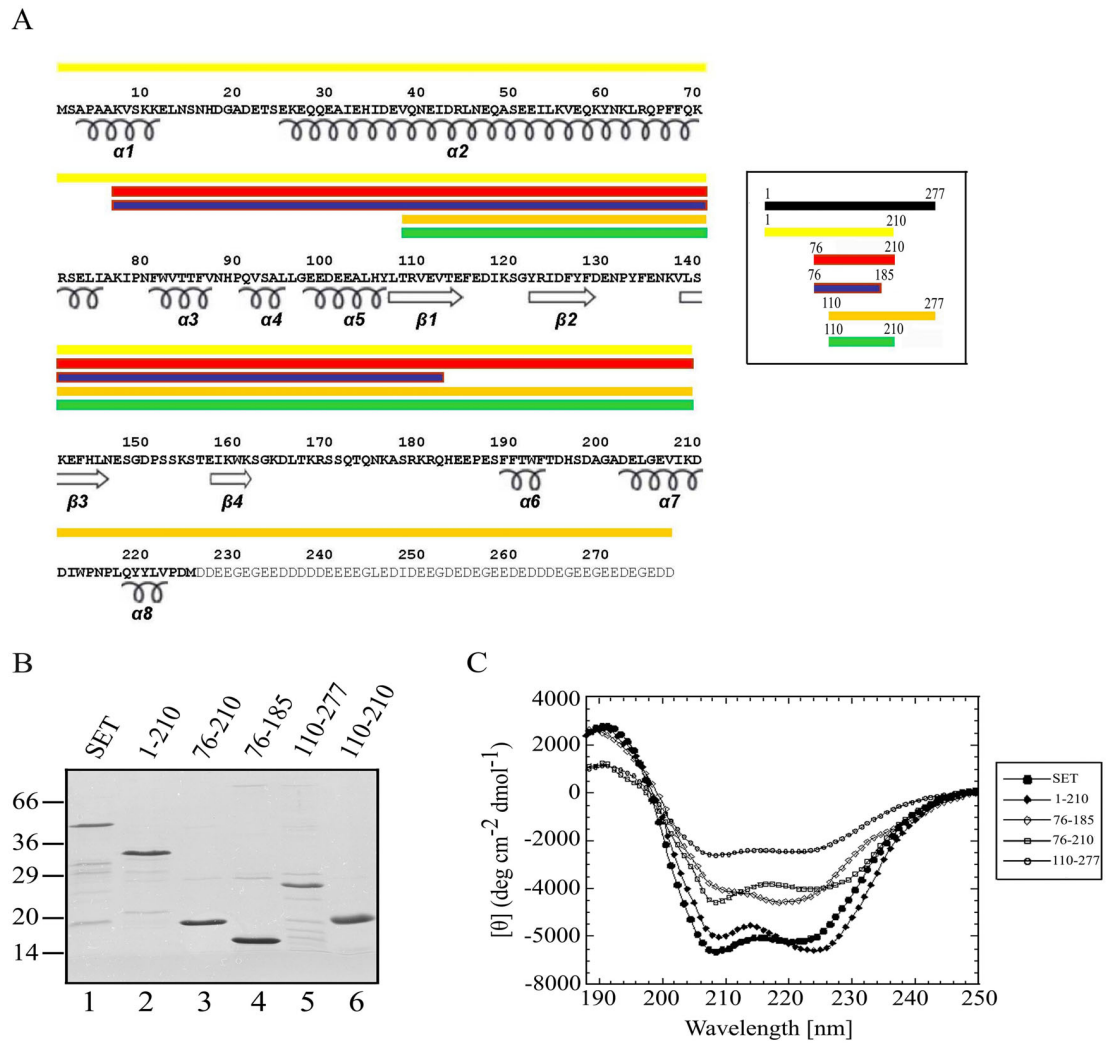
**Design and structural integrity of SET/TAF-Iβ deletion constructs**. (A) Aminoacid sequence and secondary structure elements of SET/TAF-1β (PBD code 2E250) and its deletion mutants; SET/TAF-Iβ (1–210) (yellow), SET/TAF-Iβ (76–210) (red), SET/TAF-Iβ (76–185) (blue), SET/TAF-Iβ (110–277) (orange), SET/TAF-Iβ (110–210) (green)(insert). Residues in the acidic stretch are shown in grey. (B) Wild type SET/TAF-Iβ and its deletion mutants were expressed and purified as GST-tagged proteins and subsequently the GST moiety was removed, as indicated in the Methods section. The proteins were analysed by SDS-PAGE and Coomassie blue staining. (C) Circular dichroism spectra of wild type SET/TAF-Iβ (1–277) (filled hexagon), SET/TAF-Iβ (1–210) (filled diamond), SET/TAF-Iβ 110–277 (open hexagon), SET/TAF-Iβ 76–185 (open diamond) and SET/TAF-Iβ (76–210) (open square). Stock solutions of SET/TAF-IB polypeptides were in 50 mM Tris-HCl pH 7.5, 150 mM NaCl, 0.5 mM DTT, 1 mM EDTA in a concentration range of 10–75 μM.

Since H3 was identified as the preferred histone substrate of *SET/TAF-Iβ*, we proceeded to identify the regions of *SET/TAF-Iβ *involved in H3 binding. Based on sequence comparisons between human SET/TAF-Iβ and members of the Nucleosome Assembly Protein (NAP) family, in particular NAP-1 from yeast, whose three-dimensional structure was recently solved [12] (PDB ID: 2AYU), we designed several deletion mutants of SET/TAF-Iβ, shown in Fig. 2A. Our specific aim in the design of the mutants was to challenge the role in H3 binding of (i) the dimerisation domain, (ii) the acidic C-terminal tail and (iii) the central region of the protein. Previous structural and biochemical studies have underlined the essential role of the central region of NAP1 proteins in the interaction with histones; the β-sheet subdomain, which is part of this region, is structurally conserved in otherwise unrelated in sequence and structure histone chaperones and is believed to form a scaffold onto which histone binding elements are built [12].

Wild type SET/TAF-Iβ and its mutants were expressed and purified as GST-tagged proteins and subsequently the GST moeity was removed, as indicated in the Methods section (Fig. 2B). The structural integrity of wild-type and mutant forms of SET/TAF-Iβ was explored by Circular Dichroism Spectroscopy (CD). All constructs tested, with the exception of the polypeptide spanning residues 110–210 of the protein, were found well folded (Fig. 2C). The CD spectrum of full-length SET/TAF-Iβ is indicative of a predominantly α-helical protein. The proportion of the two main types of secondary structure varies significantly between the different mutants, depending on whether the N-terminal α-helical part or the C-terminal disordered region is included in each construct. It should be noted at this point that the C-terminal acidic stretch, the only part of the protein expected to be completely disordered in solution, was not analysed by CD due to technical problems during its purification.

**Figure 3:**
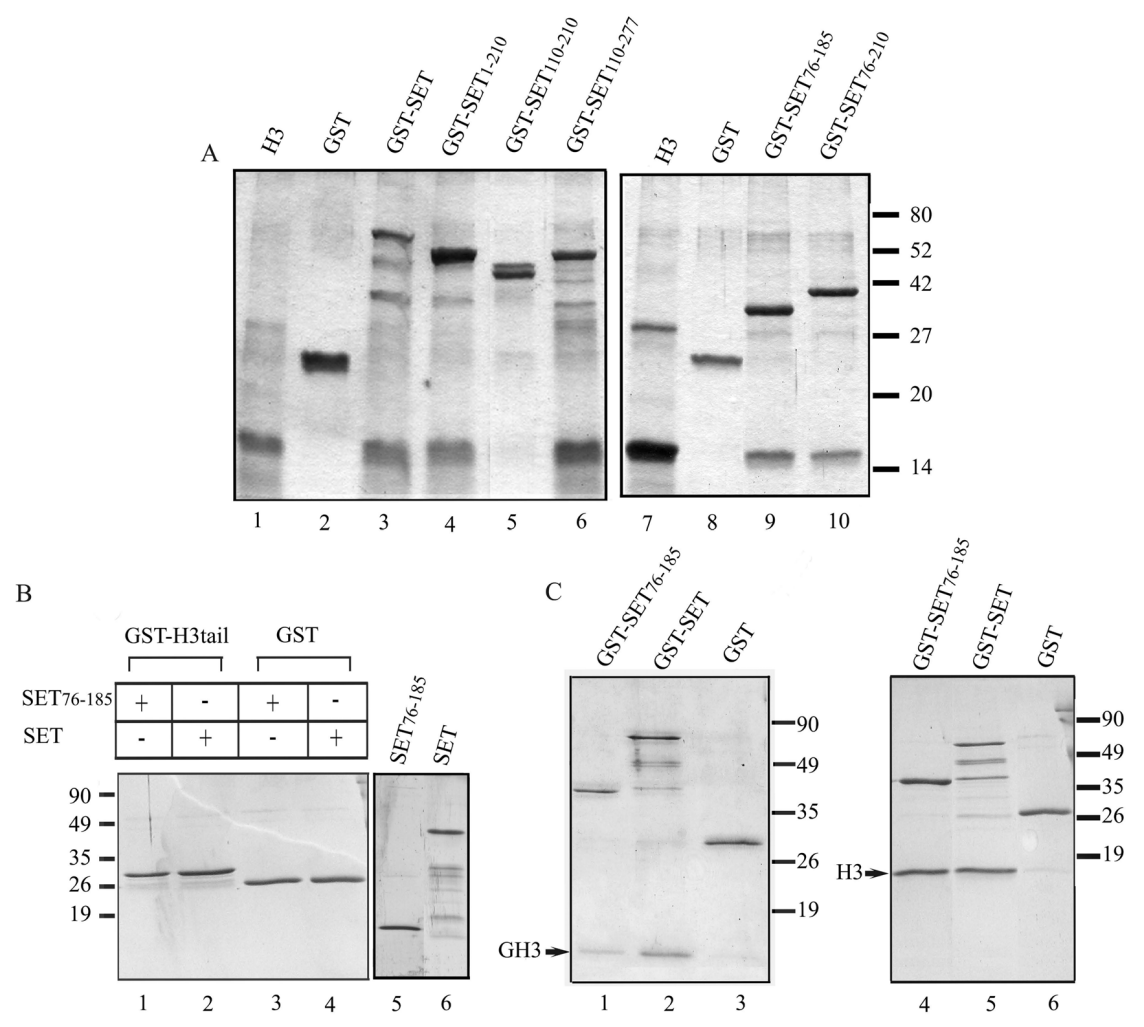
**Binding of SET/TAF-Iβ proteins to histone H3**. (A) GST-SET/TAF-Iβ (lane 3), GST-SET/TAF-Iβ constructs (lanes 4, 5, 6, 9, 10) and purified GST (lanes 2, 8) (4 μg each) were immobilised on glutathione-agarose beads and incubated with 4 μg of histone H3. The beads were washed and analysed by SDS-PAGE and Coomassie blue staining. Lanes 1 and 7 indicate the input of histone H3. (B) GST-H3 tail (lanes 1,2) or GST (lanes 3, 4) (2 μg each) were bound on glutathione-agarose beads and incubated with 4 μg of SET/TAFI-β (76–185) (lanes 1, 3) or wild type SET/TAFI-β (lanes 2, 4). The beads were washed and analysed by SDS-PAGE and Coomassie blue staining. The input of SET/TAFI-β and SET/TAFI-β (76–185) is shown in lanes 5, 6. (C)GST (lanes 3, 6), GST-SET/TAF-Iβ (lanes 2, 5), GST-SET/TAF-Iβ (76–185) (lanes 1, 4) (2 μg each) bound on glutathione beads were incubated with 4 μg of purified H3 globular domain (lanes 1, 2, 3) or recombinant histone H3 (lanes 4, 5, 6). Bound proteins were eluted with 20 μl of reduced glutathione and samples were analysed by SDS-PAGE and Coomassie blue staining.

In order to test the binding activity of SET/TAF-Iβ fragments for histone H3, we first carried out series of GST-pull down experiments with all GST-tagged mutants and H3. As shown in Figure 3A, all SET/TAF-Iβ fragments had the ability to interact with H3, with the exception of the polypeptide SET/TAF-Iβ 110–210, which was found misfolded and aggregated. These results suggest that the binding site of SET/TAF-Iβ for H3 resides mainly within its central region, despite the fact that the protein possesses a C-terminal region of pronounced negative charge. Moreover, it appears that dimerisation of SET/TAF-Iβ is not essential for the interaction, since mutants lacking the complete N-terminal dimerisation domain (residues 1–76) do not fail to bind H3 (Fig. 3A, lanes 6, 9, 10).

To assess the relative affinities of the isolated SET/TAF-Iβ deletion mutants for H3 (Fig. 2B), we resorted again to fluorescence-based titration experiments. All SET/TAF-Iβ mutants have one or more tryptophan residues in their sequence which could function as intrinsic fluorescence chromophores. The results are consistent with the pull down assays, supporting further the importance of the central region of SET in its interaction with H3. They show that, in general, shorter constructs centered around the central region of SET/TAF-Iβ (amino acids 76–185) and lacking either the N-terminal dimerisation domain and/or the acidic C-terminal tail are sufficient for H3 binding and their affinities for H3 are comparable to that of the full length protein (summarized in Table 1). The homologous region of yeast NAP1 has been shown to be crucial for the interaction with histones and has been termed "protein interaction domain" [12]. Besides the length of the mutants able to bind H3, the values of K_d_'s of most complexes and the stoichiometry deduced from the binding curves, clearly indicate that neither dimerisation nor the acidic tail-domain of SET/TAF-Iβ are absolutely necessary for the interaction (for example, compare the K_d_'s of the complexes H3-SET/TAF-Iβ (1–277) and H3-SET/TAF-Iβ (76–210) (Table 1). Our data are in agreement with previous observations on the 1:1 stoichiometry for yeast NAP1/H3 interaction [41].

We next attempted to identify the region of histone H3, which is involved in its binding with SET/TAF-Iβ. To this end, GST-fused recombinant H3 tail was tested for its abilitity to bind wild type and SET/TAF-Iβ (76–185) in pull down assays. Figure 3B shows that no binding to the amino terminal tail of H3 was detected. In contrast, binding of both proteins to the globular domain of H3 was observed (Fig. 3C, lanes 1, 2). These results suggest that the H3 N-terminal tail is not required for its interaction with SET/TAF-Iβ. On the contrary, they indicate that binding determinants of the SET/TAF-Iβ/H3 interaction lie within the globular domain of H3. This finding contradicts a previous study showing direct binding of SET/TAF-Iβ to the H3 N-terminal tail, which is disrupted when the tail is modified [34]. Discrepancies between the two studies might result from differences in the assays and the H3 fragments used. We tested full-length, native histone H3 with wild type SET/TAF-Iβ and its deletion mutants in our binding assays (Figs. 1A, 1B, 3A). We also used recombinant forms of full length H3 (Fig. 3C, lanes 4, 5), the globular part of H3 and its N-terminal tail (H3 residues 1–46). On the contrary, in the above study, SET/TAF-Iβ binding to H3 was tested using short synthetic peptides corresponding to residues 1–16 of H3, immobilized on columns [34]. Our data clearly show that the binding of SET/TAF-Iβ to H3 occurs with native H3 (Figs. 1A, 1B, 3A), which is heavily modified (S. Liokatis and A.S. Politou, unpublished results) and with recombinant forms of intact H3 and of its core region (Fig. 3C), which are devoid of any post-translational modifications. On the other hand, the binding is abolished when unmodified H3 tail is used in the pull-down assays (Fig. 3B). Therefore, neither the amino-terminal tail nor any modifications (either in the core or in the tail) of H3 seem to play an essential role in its interaction with SET/TAF-Iβ.

## Discussion

The histone binding studies described in this work were made with wild-type and SET/TAF-Iβ deletion mutants. These mutants were designed on the basis of its sequence similarity to NAP-1 from yeast, whose three-dimensional structure was known [12], since the structure of SET/TAF-Iβ had not been determined yet. While this manuscript was in preparation, the crystal structure of SET/TAF-Iβ was reported (PDB ID: 2E50) [51]. The crystal structure shows that SET/TAF-Iβ forms a dimer that assumes a headphone like shape, with each subunit consisting only of an unstable and highly mobile α-helix, a long backbone helix, and an α+β "earmuff domain" (amino acids 1–24, 25–78, and 79–225, respectively). The two backbone helices are not arranged in a coiled-coil fashion, but interact hydrophobically in an antiparallel manner to form the dimer. The earmuff domain is attached to the concave side of the backbone helix and its lower part is highly mobile in aqueous solution. Comparison of the crystal structure of SET/TAF-Iβ with that of NAP-1 revealed that the two proteins exhibit the highest similarity in the region of the earmuff domain. Inspection of the structure itself and biochemical analysis of the wild-type protein and of 18 triple mutants led to important conclusions regarding the histone and DNA binding properties of SET/TAF1β, which provide insight into its histone chaperone function. More specifically, Muto *et al*. [51] have shown that: (a) SET/TAF1β has a histone chaperone activity specific to H3/H4, (b) its acidic C-terminal stretch is not important for H3/H4 binding and (c) the bottom part of the earmuff domain, formed mainly by the β-structure, which is exposed to the solvent, is more likely responsible for its histone chaperone activity. No affinity measurements of the tested interactions have been reported in that work.

Our approach, based on the design of deletion mutants and functional domains of SET/TAF1β and on the quantitative comparison of their affinities for histones, allowed us to draw conclusions that extend and complement this study [51] by shifting the focus towards affinity differences of isolated histones for the SET/TAF1β chaperone.

**Figure 4:**
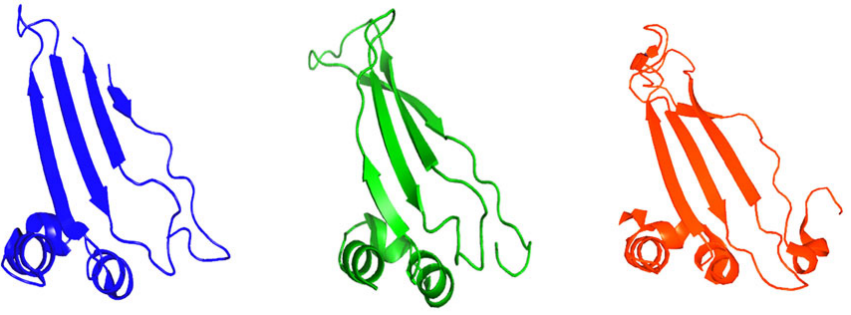
**Mapping on the SET/TAF-Iβ structure of the central region essential for H3 binding**. Views of the central region of SET/TAF-Iβ found to be essential for H3 binding in this work (*blue*), the homologous domain (named subdomain C) of yeast NAP-1 (*green*) and the homologous domain of yeast Vps75 (*red*). PDB codes used are 2E50 for human SET/TAF-Iβ, 2AYU for yeast NAP-1 and 2ZD7 for yeast Vps75. The central β-sheet is a common structural motif found in all histone chaperones known todate. Molecular images were generated with PyMol [61].

When the domains analysed in this work are mapped onto the SET/TAF-Iβ sequence it becomes clear that the central region of SET/TAF-Iβ, spanning residues 76–185 in the earmuff domain, is crucial for histone binding. The sequence of this particular region is extremely conserved between human, mouse and *Xenopus *SET/TAF-Iβ and its tertiary structure is very similar to the homologous region in yeast NAP-1 (Fig. 4). It has been recently shown that the amphipathic β-sheet formed in this region represents a structural motif common to all histone chaperones with known structures (NAP-1, nucleoplasmin, ASF1, CAF-1), despite a wide variation in sequence, structural context and overall architecture of the various chaperones [12,13]. These observations suggest a strong evolutionary conservation and underscore the relevance of this motif for the common function of histone chaperones with their differences accounting for distinct roles in chromatin assembly. Moreover, our data provide concrete and quantitative evidence that removal of the acidic C-terminal tail of SET/TAF-Iβ does not affect its affinity for histone H3. It can be easily inferred from this observation that charge neutralization between the basic histones and acidic chaperones is dispensable for their interaction, a conclusion further supported by the complete absence of acidic stretches in certain chaperones, such as ASF1 [52]. It cannot be excluded, though, that post-translational modifications of the acidic stretch could have an effect on the interaction between chaperones and their respective histone substrates *in vivo*. Acidic stretches of NAP1 have been shown to be highly polymorphic in a variety of tissues and organisms due to various degrees of polyglutamylation [12], a modification that could significantly affect the charge and physicochemical properties of SET/TAF-Iβ in a reversible manner and lead to further refinement of its interaction with histones *in vivo*.

Very recently three different reports on the structure and biochemistry of a new member of the histone chaperone family, Vps75 from yeast [53], revealed a remarkable structural similarity with SET/TAF-Iβ and, in agreement with our observations, highlighted the importance of the central cleft for histone binding [54-56]. Comparison of the high resolution structures of Vps75 and SET/TAF-Iβ clearly shows that the two proteins share, besides the overall topology, several other structural features, such as the lack of the extended N-terminal tail and of the accessory domain identified in NAP-1, which point to Vps75 as the closest known structural homologue of SET/TAF-Iβ in yeast (Fig. 4). Biochemical experiments with wild-type and mutated Vsp75 show that its central cleft is important for histone binding and for its interaction with the histone acetyltransferase Rtt109. Significant functional similarities with SET/TAF-Iβ include the predominantly nuclear localization, the involvement in non-canonical histone chaperone activities, the interaction with both H2A/H2B and H3/H4, the irrelevance of the H3 tails for this interaction and the lack of H2A/H2B dimer stripping activity, in sharp contrast to NAP-1 which readily removes H2A/H2B dimers from assembled nucleosomes [55]. There are, however, notable differences between the two proteins, especially in the central cleft size and the intrinsic motion of this part resulting in altered charge density and surface electrostatic potential, that have been correlated with the unique function of Vps75 to interact with Rtt109 for histone acetylation, but also with likely different histone substrate specificities [54,56]. Evidence to this direction is provided by our quantitative binding studies, which indicate a clear preference of SET/TAF-Iβ for H3 over H2B binding, unlike Vps75 that has been shown to preferentially interact with H2A/H2B [55]. In addition, the histone chaperone activity of Vps75 has been found to be intimately linked with stimulation of H3 acetylation through the formation of the multifunctional Vps75-Rtt109 complex that acetylates H3 and deposits H3/H4 onto DNA [54]. Affinity measurements for the interaction of H3 with wild-type and Vps75 mutants previously reported to be defective in histone binding and chaperone activity of SET/TAF-Iβ [51] demonstrated that the region of Vps75 responsible for Rtt109 activation is found in the acidic cleft of Vps75, but is not the one involved in histone binding [54]. This region corresponds to the stretch ^209^KDD^211 ^of SET/TAF-Iβ which maps beyond the part we identified as mainly responsible for the interaction with H3. Overall, it appears that SET/TAF-Iβ, NAP-1 and Vps75, although structurally very similar, are functionally distinct members of the NAP family of histone chaperones.

## Conclusion

The quantitative study of the histone-binding properties of SET/TAF-Iβ described here and its dissection in structural/functional domains were based on a type of analysis, that has not been extensively used in the chromatin field, but has allowed us to draw several important conclusions regarding its histone chaperone function. In short, our data showed that SET/TAF-Iβ binds preferentially to histone H3, mainly through its central region, while the dimerisation domain and the acidic tail, both hallmarks of histone chaperones, are not essential for the interaction. On the other hand, binding of H3 to SET/TAF-Iβ, is mediated by its core region and does not seem to depend on the presence or the modification status of the H3 amino-terminal tail. Our findings regarding the ability of distinct SET/TAF-Iβ domains to bind histones are in good agreement (i) with inferences made by close inspection of the tertiary structure, (ii) with the previously deduced 1:1 stoichiometry for the SET/H3 interaction, (iii) with recent suggestions on the relevance of the central β-sheet motif for the common function of histone chaperones [12] and (iv) with the importance of affinity differences for histone chaperone activity [45,48]. Differences of SET/TAF-Iβ with other histone chaperones, that could account for a distinct function, include the clear preference for H3 binding, the irrelevance of the H3 tails or their modification status for the interaction and the inability to bind linker histone H1, together with the recently reported lack of H2A/H2B dimer stripping activity [49,50].

## Methods

### Expression and purification of recombinant proteins

SET/TAF-Iβ and fragments of SET/TAF-Iβ were subcloned into pGEX vectors (Pharmacia) in frame with glutathione S-transferase (GST). GST-proteins were expressed in BL21 cells and purified from lysates according to standard procedures [57]. To remove the GST moiety, GST-proteins were immobilized on glutathione beads and digested with the PreScission Protease (Amersham Pharmacia Biotech), 2 units of enzyme/100 μg of bound GST-protien in 20 μl cleavage buffer (50 mM Tris-HCl, pH 7.0, 150 mM NaCl, 1 mM EDTA, 1 mM DDT, 0.1% NP40) at 4°C for 12 h. Linker histone H1 and core histones H2A, H2B, H3 and H4 were obtained from Roche. A pET3a expression vector carrying the cDNAs encoding H3 and histone H3 globular domain (aa 27–135) from *Xenopus laevis *was a kind gift from K. Luger, University of Colorado (California, USA). Recombinant H3 and tailless H3 were expressed in *E. coli *BL21 (DE3) cells and purified under denaturing conditions using SP-sepharose chromatography, as previously described [58]. After removal of urea with extensive dialysis, the samples were lyophilized, dissolved in water and their concentration was adjusted to 1 mg/ml. The yeast H3 tail region (1–46) was expressed as a fusion protein with a N-terminal GST tag from a pGET2T expression vector kindly provided from M. Grunstein, University of California and was purified according to standard procedures [59]. Pull-down assays were performed with fresh preparations of all histone constructs.

### H3/H4 tetramer assembly

H3/H4 tetramer reconstitution was based on Luger et al, 1997 [1], with slight modifications. Native H4 was purchased from Roche and dissolved in water at a concentration of 1 mg/ml. Recombinant H3 was prepared as described above. The two proteins were mixed at equimolar ratio and dialyzed against unfolding buffer (6 M Urea, 20 mM Tris-HCl pH 7.5, 5 mM β-mercaptoethanol) overnight. Tetramer assembly was achieved using the salt-dialysis method as follows: The histone mixture was dialyzed against at least three changes of refolding buffer (2 M NaCl, 10 mM Tris-HCl pH 7.5, 1 mM EDTA, 5 mM β-mercaptoethanol). Then, the concentration of NaCl was adjusted by dialysis to 1 M, 0.5 M, 0.15 M for 5 h, except the last dialysis step which was left overnight. Precipitated material was removed by centrifugation and the reconstituted tetramer was tested directly in pull-down assays.

### GST-pull down assays

Fusion proteins (approximately 2–3 μg each) were immobilized on glutathione-Sepharose beads and incubated with 4 μg of histones (H1, H2A, H2B, H3, H4) in TNMT buffer (20 mM Tris-HCl, pH 7.5, 0.5 M NaCl, 2 mM MgCl_2 _and 1% Triton X-100) in a total volume of 0.25 ml. Reactions were carried out for 60 min at room temperature. The beads were harvested, washed three times and resuspended in 25 μl of SDS sample buffer. For pull-down assays using (H3/H4)_2 _tetramers, GST-SET/TAF-Iβ and GST were immobilized on glutathione-agarose beads in the washing buffer (20 mM Tris-HCl pH 7.5, 150 mM NaCl, 5% sucrose, 0.1% NP-40, 1 mM EDTA, 1 mM DTT, 1 mM PMSF). After washing the beads twice, H3/H4 tetramer was added and incubated for 1 h in the assay buffer (20 mM Tris-HCl pH 7.5, 300 mM NaCl, 5% sucrose, 0.1% NP-40, 1 mM EDTA, 1 mM DTT, 1 mM PMSF, at room temperature. The beads were washed five times with the assay buffer and once with the washing buffer. Bound proteins were eluted with hot SDS-sample buffer and samples were analysed on 13% SDS-polyacrylamide gels and detected by Coomassie Blue staining.

### Fluorescence Spectroscopy Binding Assays

Binding of the various SET/TAF-Iβ constructs with histones was followed by monitoring the intensity of the fluorescence emitted by the tryptophan residues of SET/TAF-Iβ polypeptides upon addition of increasing amounts of calf thymus histone H3 [46,47]. A Hitachi F-2500 fluorescence spectrophotometer, fitted with a thermostatically controlled jacketed cell holder and interfaced with a Neslab RTE-111 water-bath was used. Fluorescence emission spectra in the range 300–400 nm were recorded with an excitation wavelength of 295 nm. SET/TAF-Iβ polypeptides at several initial concentrations ranging from 0.2 to 4 μM were titrated with increasing amounts of purified calf thymus H3 stock solution in PBS buffer. All measurements were performed at 25°C in a quartz cell with path length of 1 cm (Hellma) under constant stirring. After each ligand addition, the samples were left to equilibrate for 10 min before the equilibrium fluorescence readings were recorded. The dissociation constant (*K*_*d*_) for the complexes formed was estimated by non-linear regression analysis (KaleidaGraph, Synergy Software) relating the change in SET fluorescence intensity at 345 nm (corrected for buffer contribution and for dilution) to the total added histone concentration through Equation 1:

(1)

where F, *F*_0_, and *F*_max _are the fluorescence intensities measured at each titration point, at zero ligand concentration and at saturation, respectively, and [*L*] is the concentration of free ligand calculated by solving the quadratic equation

(2)

where [P]_0 _and [*L*]_0 _are the protein and total ligand concentrations added, respectively. Each titration was repeated three times.

### Circular Dichroism

Circular dichroism spectra in the Far UV range (180–260 nm) were recorded on a Jasco J-815 spectropolarimeter interfaced with a Peltier element for temperature control. The instrument was calibrated with a 0.1% aqueous solution of *d*-10-camphor sulfonic acid. Stock solutions of SET/TAF-IB polypeptides were in 50 mM Tris-HCl pH 7.5, 150 mM NaCl, 0.5 mM DTT, 1 mM EDTA and their concentration ranged from 10 to 75 μM. Spectra were recorded at 25°C, with 0.2 nm resolution, averaged over five scans and were baseline corrected by subtraction of the buffer spectrum at the same temperature. Quartz cells with path lengths of 0.02 or 0.1 cm were used (Hellma). The combined absorbance of cell, sample and solvent was kept at < 1 over the measured range. Secondary structure was estimated as described previously [60]. Molecular images were generated with PyMol [61].

## Authors' contributions

ZK, AE carried out cloning, purification of wild-type and mutant proteins and performed fluorescence spectroscopy-based binding assays. IS, SL carried out the tetramer assembly, purification of wild-type and mutant proteins and GST-pull down assays. EN participated in the design and coordination of the study. AP and TP designed the study, supervised data collection and analysis and drafted the manuscript. All authors read and approved the final manuscript.
